# Multiple caecal granular cell tumours—a case report

**DOI:** 10.1093/jscr/rjab396

**Published:** 2021-09-22

**Authors:** Kar Yin Fok, Chow Heok P’Ng, Hema Mahajan, Martijn Pieter Gosselink, Toufic El-Khoury

**Affiliations:** Department of Colorectal Surgery, Westmead Hospital, Westmead, New South Wales, Australia; Department of Pathology, Westmead Hospital, Westmead, New South Wales, Australia; Department of Pathology, Westmead Hospital, Westmead, New South Wales, Australia; Department of Colorectal Surgery, Westmead Hospital, Westmead, New South Wales, Australia; Department of Colorectal Surgery, Westmead Hospital, Westmead, New South Wales, Australia

## Abstract

Granular cell tumours (GCTs) are generally benign neoplasms, which are believed to be of neural origin. They are uncommon in the gastrointestinal tract. They are rarely found in the colon and even more rarely found to be multiple. We present a case of a man who underwent a right hemicolectomy for a submucosal lesion and polyps and was found to have multiple nodules diagnosed as caecal GCTs with cellular atypia. While uncommon, this case shows it remains an important differential due to implications for patient management, given the often benign nature of disease.

## INTRODUCTION

Granular cell tumours (GCTs) are generally benign neoplasms believed to be of neural origin. They are uncommon in the gastrointestinal tract and are rarely found in the colon and are rarely multiple. They are often diagnosed incidentally during endoscopic or imaging investigation. Here, we present a case of multiple colonic GCTs diagnosed during workup of colonic polyps and a submucosal lesion.

## CASE REPORT

A 46-year-old man from Papua New Guinea underwent colonoscopy for investigation for intermittent rectal bleeding. There were no associated weight loss or altered bowel habits; however, he had a history of hyperplastic polyps on a previous colonoscopy 7 years prior. There was also a family history of colorectal cancer, which affected his father and paternal aunt in their 60s and his maternal uncle in his 40s. He had a 30-pack-year smoking history, but aside from cluster headaches, had no other medical or surgical history and was not on any regular medications.

His colonoscopy revealed multiple polyps, 12 in total, throughout the colon. They were serrated or hyperplastic in appearance, ranged from 4 to 10 mm, were sessile and all were removed via cold snare. In the caecum, there was a 10 × 10 mm hard submucosal lesion. This differed in appearance to two smaller yellow submucosal lesions nearby, which were soft, and appeared to be lipomatous. Deep biopsies were taken of this hard submucosal lesion in the caecum. It was not amendable to endoscopic mucosal resection due to risk of perforation.

He was referred by his gastroenterologist to a colorectal surgeon for consideration of excisional biopsy. On computed tomography (CT) of abdomen and pelvis, there was no evidence of metastasis or advanced local spread of tumour. Deep biopsies of the caecal lesion were normal, and other polyps were hyperplastic. He was suspected of having a hyperplastic polyposis syndrome. Differentials for the caecal lesion, however, included gastrointestinal stromal tumour (GIST), lipoma or carcinoid tumour. Serum serotonin, chromogranin A as well as 24-h urinary 5-HIAA were all within normal limits. After discussion with the patient, a plan for laparoscopic right hemicolectomy was made.

Intraoperatively, caecal polyps were noted, including firm nodules. There were no complications from his surgery, and his post-operative stay was unremarkable. He was discharged home after opening bowels and tolerating a light diet.

**Figure 1:**
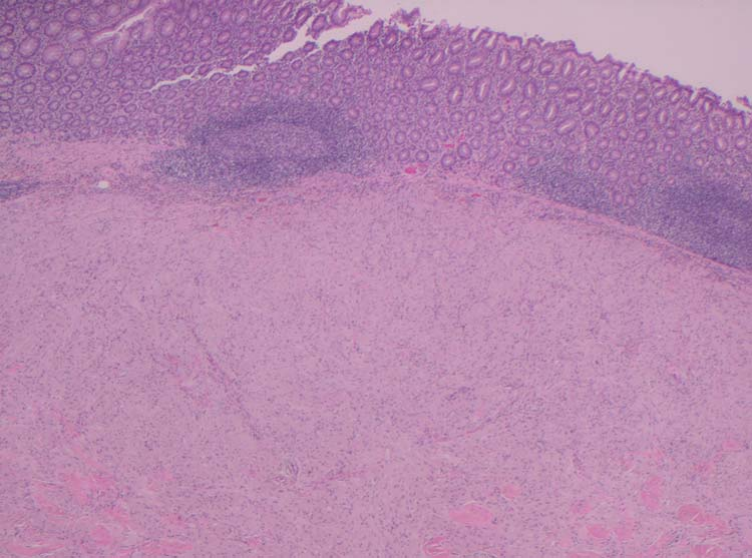
H&E stain magnification × 2.

**Figure 2:**
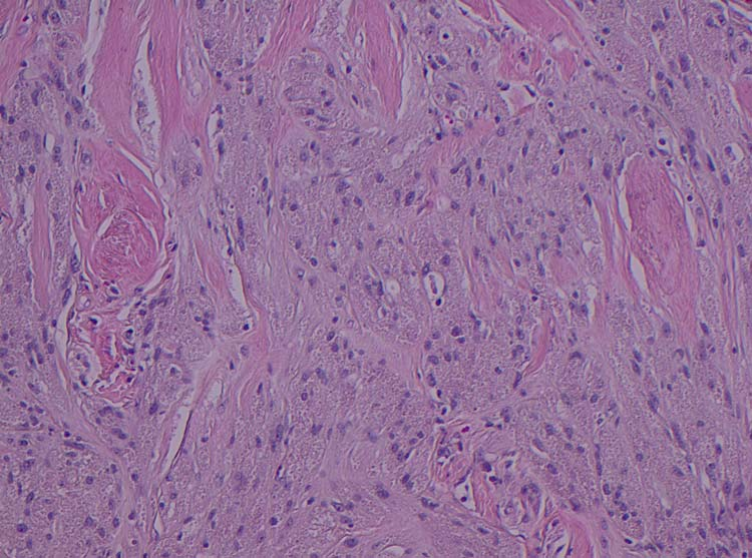
H&E stain magnification × 4.

**Figure 3:**
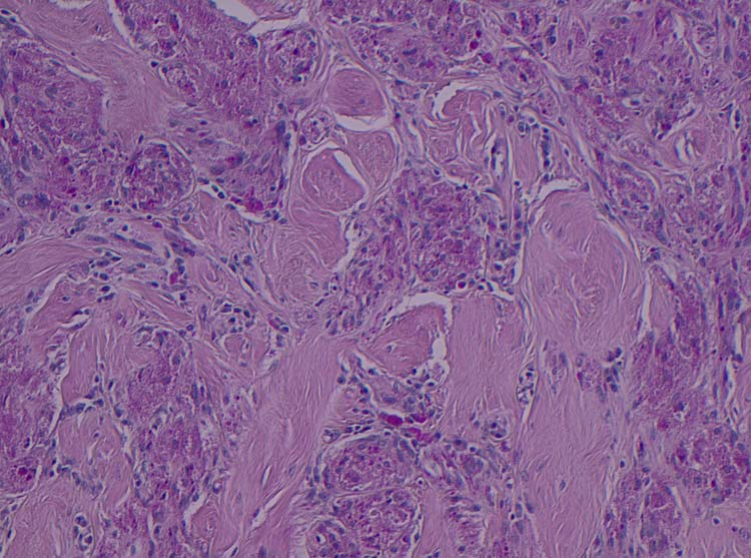
PAS stain showing PAS granular cytoplasm, magnification × 4.

**Figure 4:**
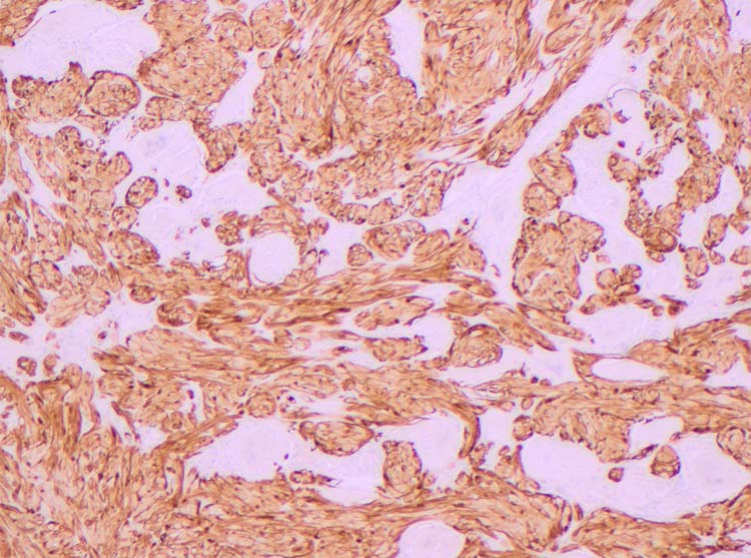
S-100 stain positive, magnification × 4.

Histopathology of the right hemicolon found five firm nodules in the caecum and an additional seven polyps in the ascending colon macroscopically. Microscopically, a total of eight lesions in the caecum and ascending colon were found to be GCTs, varying in size from 1 to 7 mm. They were submucosal with intact covering mucosa (Figs 1 and 2). Tumour cells had periodic acid-Schiff (PAS)-positive granular cytoplasm and small nucleoli (Fig. 3). Immunohistochemical staining was positive for S-100 (Fig. 4) and inhibin. Twenty-four lymph nodes identified showed no abnormality. Spindling was seen in some tumours in this case, but overall, there are less than three worrisome features, and as such, these GCTs were considered atypical. Other polypoid lesions identified included two hyperplastic polyps and a submucosal lipoma.

He remained well at 3-week follow-up post-surgery.

## DISCUSSION

GCTs were first described by Abrikossoff [1] who observed a tongue lesion thought to originate from muscle. Subsequent literature, with aid of immunohistochemistry, has described GCTs as being of neural origin, in particular, Schwann cell differentiation [2]. GCTs are almost always benign, with only 1–2% of reported cases being malignant [3].

Histologically GCTs are characterized by plump neoplastic cells with abundant eosinophilic granular cytoplasm. Immunohistochemistry for neural markers, S-100 protein and NSE are diffusely positive, while other markers include inhibin, calretinin and nestin [2, 4]. Fanburg-Smith *et al*. [3] described microscopic criteria to predict malignant potential, where GCTs meeting three or more criteria were classified as histologically malignant.

GCTs are typically found in the skin, tongue and subcutaneous tissues, particularly on the chest and upper extremities. They are uncommon within the gastrointestinal tract, accounting for 5–9% of cases [5]. Within this subgroup, they are preferentially found in the oesophagus, up to three quarters of all gastrointestinal GCTs, while colorectal and perianal GCTs are rarer in comparison [6].

Colorectal GCTs are asymptomatic, usually found incidentally on colonoscopy or imaging [7]. Endoscopically, GCTs tend to appear as solitary small sessile polyps, or submucosal lesions. Colonic GCTs have a predilection for being located in the caecum and ascending colon and rectum.

A case series, in 2010, described 26 cases of colorectal GCT from 1995 to 2009, with an equal gender distribution [2]. The majority of colorectal GCTs were found in the right colon (19 of 26 patients, 73%), supported by Na *et al*. [7] (19 of 30 patients, 63%) in their case series. Na *et al*. [7], however, showed male predominance (22 of 27 patients, 81%). Colorectal GCTs were also more likely to have larger nuclei, display an infiltrative growth pattern, with increased nuclear atypia, compared to other gastrointestinal GCTs, although these features were not necessarily related to malignant behaviour.

Multiple GCTs are uncommon and are estimated to occur in ~4–16% of patients [2]. Melo *et al*. [8] had reported a case of 52 GCTs within the same patient, throughout the gastrointestinal tract. For colonic GCTs, Yamada *et al*. [9] reported a case of eight GCTs in the ascending colon, while Rossi *et al*., in 2000, reported a case of multiple GCTs diagnosed via screening colonoscopy for family history in addition to the 15 prior cases of multiple colonic GCTs in the literature at the time [10]. Saleh *et al*. [11] reported a case with multiple GCTs involving colon, appendix and also the mesentery. There is suggestion that multiple GCTs may be associated with syndromes, such as neurofibromatosis and Noonan syndrome, however, there is evidence to support this currently, especially in the colorectal setting.

Due to the rarity of the condition, there is limited evidence on the management of colonic GCTs. In general, excision with close surveillance endoscopy, serial endoscopic mucosal removal or surgical excision have been described due to the relatively benign nature of the disease.

In summary, we present a case of multiple GCTs in the caecum and ascending colon in a patient who underwent right hemicolectomy for submucosal lesion, polyps and family history of colorectal cancer. Although rare, it remains a differential to keep in mind.

## CONFLICT OF INTEREST STATEMENT

None declared.
